# Acetylation of the Cell-Fate Factor Dachshund Determines p53 Binding and Signaling Modules in Breast Cancer

**DOI:** 10.18632/oncotarget.1094

**Published:** 2013-06-21

**Authors:** Ke Chen, Kongming Wu, Michael Gormley, Adam Ertel, Jing Wang, Wei Zhang, Jie Zhou, Gabriele DiSante, Zhiping Li, Hallgeir Rui, Andrew A. Quong, Steven B. McMahon, Haiteng Deng, Michael P. Lisanti, Chenguang Wang, Richard G. Pestell

**Affiliations:** ^1^ Department of Cancer Biology, Thomas Jefferson University, Philadelphia, PA, USA; ^2^ Kimmel Cancer Center, Thomas Jefferson University, Philadelphia, PA, USA; ^3^ Proteomics Resource Center, Rockefeller University, New York, NY, USA

**Keywords:** p53, breast cancer, cell fate, stem cells, dach

## Abstract

Breast cancer is a leading form of cancer in the world. The *Drosophila Dac* gene was cloned as an inhibitor of the hyperactive epidermal growth factor (EGFR), *ellipse*. Herein, endogenous DACH1 co-localized with p53 in a nuclear, extranucleolar compartment and bound to p53 in human breast cancer cell lines, p53 and DACH1 bound common genes in Chip-Seq. Full inhibition of breast cancer contact-independent growth by DACH1 required p53. The p53 breast cancer mutants R248Q and R273H, evaded DACH1 binding. DACH1 phosphorylation at serine residue (S439) inhibited p53 binding and phosphorylation at p53 amino-terminal sites (S15, S20) enhanced DACH1 binding. DACH1 binding to p53 was inhibited by NAD-dependent deacetylation via DACH1 K628. DACH1 repressed p21^CIP1^ and induced RAD51, an association found in basal breast cancer. DACH1 inhibits breast cancer cellular growth in an NAD and p53-dependent manner through direct protein-protein association.

## INTRODUCTION

Breast cancer is one of the leading causes of cancer mortality in women worldwide, with an estimated 232,340 new cases in 2013 in the United States. p53 is the most frequent target for mutation in tumors, occurring predominantly as missense mutations, several of which occur as “hot spot” mutations in the DNA-binding core domain [1]. In the cellular environment without DNA damaging or oncogenic stress, p53 is short lived. Activation of p53 in response to cellular stress contributes to the induction of cell cycle arrest, cellular senescence and apoptosis, and cellular differentiation. Missense mutations lead to the accumulation of p53 mutant protein, which in humans correlates with poor outcome in a variety of human tumors, including breast cancer [2]. The R248Q missense mutant in particular is associated with poor prognosis in breast cancer [2]. The function of p53 is modulated through altered cellular localization and post-translational modifications [3] , which recruit protein complexes to coordinate gene expression and control cellular phenotype. Understanding the mechanisms governing p53 function via its associated protein binding partners is fundamental to tumor biology.

Initially cloned as a dominant inhibitor of the hyperactive EGFR, *Ellipse*, in *Drosophila*, the mammalian DACH1 regulates expression of target genes in part through interacting with DNA-binding transcription factors (c-Jun, Smads, Six, ERα), and in part through intrinsic DNA-sequence specific binding to Forkhead binding sites [4-7]. The *Drosophila dac* gene is a key member of the retinal determination gene network (RDGN), which also includes *eyes absent (eya), ey, twin of eyeless (toy), teashirt (tsh)* and *sin oculis (so)*, that specifies eye tissue identity.

Several lines of evidence suggest DACH1 may function as a tumor suppressor. Clinical studies have demonstrated a correlation between poor prognosis and reduced expression of the cell-fate determination factor DACH1 in breast cancer [7], and loss of DACH1 expression has been observed in prostate and endometrial cancer [6, 8]. DACH1 inhibits breast cancer tumor metastasis and reduces breast cancer stem cell expansion via Sox2/Nanog [9]. Although these studies suggest DACH1 may function as a tumor suppressor, the molecular mechanisms remain poorly defined.

Given the importance of p53 as a tumor suppressor in human breast cancer, we examined the role of DACH1 in p53-mediated breast cancer cellular growth suppression. These studies identified DACH1 as a novel p53 binding protein that enhances p53 WT tumor suppressor function. DACH1 is shown to be acetylated at its carboxyl terminus, which governs p53 binding and function.

## RESULTS

### DACH1 associates with p53 in human breast cancer cells

In order to determine whether DACH1 associates with p53, we first examined the association of endogenous p53 with DACH1 using the MDA-MB-453 breast cancer cell line, which express wild-type p53. DACH1 was localized using a previously well characterized monoclonal DACH1 antibody [7], in a nuclear, extranucleolar location (S. 1A). p53 was in both nuclear and nucleolar location, and co-localized with DACH1 (S. 1A) (high resolution merged image, S. 2).

To further confirm the association between p53 and DACH1, immunoprecipitation (IP) –Western blotting (WB) was conducted using an antibody, either to p53 with sequential WB to DACH1, or immunoprecipitation with a DACH1 specific antibody and sequential WB for p53 (S. 1B). In both IP-WB approaches, p53 associated with DACH1. In order to determine the domains of DACH1 required for association with p53, the alternate spliced forms of DACH1 (DACH1b, 1c) were expressed with wild-type DACH1a in HEK 293T cells. IP-WB was conducted. WB demonstrated the presence of DACH1a, 1b, 1c (S. 1C) and IP-WB revealed DACH1 using anti-FLAG and the association with p53 using a p53 specific antibody (S. 1C). We extended these studies to examine the association between DACH1 and p53 in other breast cancer cell lines. Immunoflourescent analysis of DACH1 and p53 in MCF-7 cells demonstrated the co-localization of p53 and DACH1 in an intranuclear extranucleolar location through a merged image (S. 1D). IP-WB with an anti-FLAG antibody directed towards the amino terminal FLAG epitope of DACH1 revealed its association with p53 (S. 1E). In MDA-MB-231 cells, which have low levels of endogenous DACH1, the stable reintroduction of DACH1 under control of a ponasterone-regulated promoter demonstrated that p53 and DACH1 associated in an intranuclear extranucleolar location merged upon the induction of DACH1 by (S. 1F).

### DACH1 binding to p53 is abrogated by p53 hot spot mutations and p53 acetylation at K120

We next considered the possibility that specific residues within p53 may determine physical association with DACH1. In order to identify the domains of p53 required for binding to DACH1, a series of expression vectors were deployed encoding p53 “hot spot” point mutations identified in breast cancer. The N-terminal FLAG epitope was used to immunoprecipitate DACH1 (S. 3A). Western blot analysis demonstrated the association of endogenous p53 with DACH1. DACH1 showed wild-type binding to R175H, the mutant found in MDA-MB-231 cells [10]. The R248Q and R273H are “hot spot” mutations in breast cancer that are located in the DNA-binding domain and central core region. Binding of DACH1 to the R273H mutant was reduced 60% and the R248Q mutant was incapable of binding DACH1 (S. 3A,B).

As p53 function is regulated by phosphorylation, we examined a panel of p53 phosphorylation site mutations (S. 3C). Mutation of Serine 15 and Serine 20 reduced DACH1 binding by > 40% (S. 3C,D). Post-translational modification of p53 by acetylation contributes to p53-dependent growth arrest and apoptosis, without affecting p53-MDM2-dependent feedback [11]. p53 acetylation within the carboxyl-terminus contributes to the DNA damage response [12, 13]. p53 acetylation within the DNA binding domain at K120 selectively enhances the ability of p53 to regulate apoptosis by altering DNA binding to selective targets [14-16]. In multiple experiments, the p53 acetylation site mutant K120R showed significantly enhanced binding to DACH1 (S. 3E,F). The polymorphic residue, Proline 72, which is located in the transactivation domain 2, reduced DACH1 binding (S. 3G,H). Mutation of the carboxyl-terminal module of acetylation sites (p53 9K/R) caused no significant change in DACH1 binding (Fig. S3G,H), while the 9K/R, K120R showed enhanced binding (S. 3H). A schematic representation summarizing the residues of p53 residues, mutated in human breast cancer and the K120 site for DACH1 binding, are shown in S. 3I.

### Common genes are regulated by and bind DACH1 and p53 in the context of local chromatin

**Figure 1:**
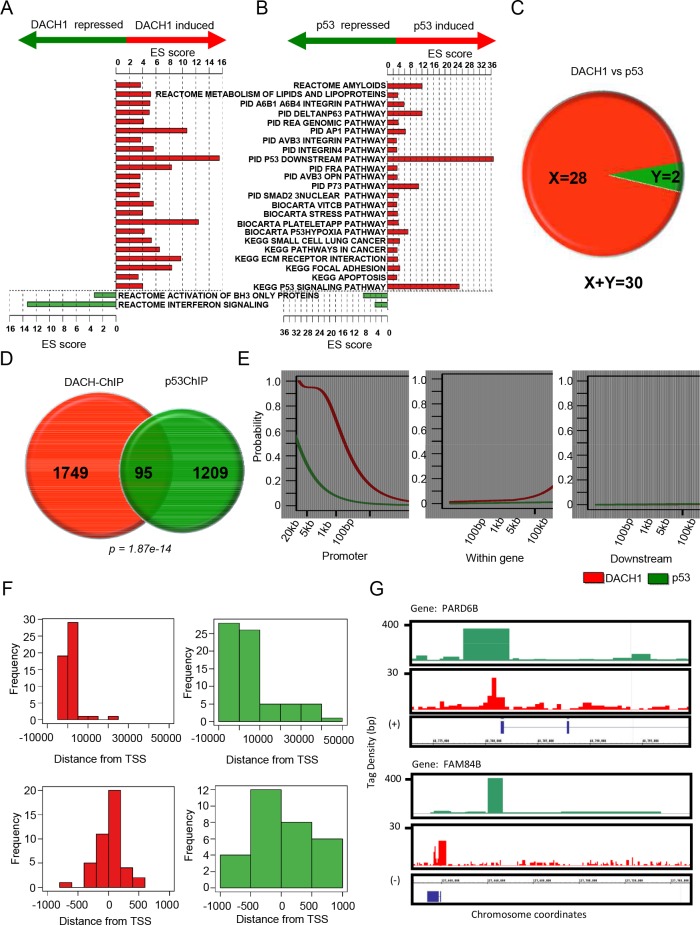
p53 and DACH1 regulate common functional gene modules and bind common genes in ChIP-Seq (A-B) Molecular pathways enriched with genes regulated by DACH1 and p53. Pathways enriched with induced genes are represented by red bars. Pathways enriched with repressed genes are represented by green bars. ES score is equal to the confidence of enrichment expressed as –log(p). (C) Number of genes co-regulated by DACH1 and p53. X = genes upregulated by both DACH1 and p53. Y = genes downregulated by both DACH1 and p53. (D) Pie diagram of overlapping genes binding DACH1 and p53 in ChIP-Seq. (E) Cumulative distribution of the location of Chip-Seq peaks with respect to neighboring genes. (F) Histogram of the location of DACH1 and p53 Chip-Seq peaks relative to the transcription start site (TSS) at -10 kb to +50 kb (upper panel) and -1 kb to +1 kb (lower panel). (G) Integrated genome browser visualization of tag density profiles for ChIP-Seq DACH1 and ChIP-Seq p53. Selected genes are PARD6B, par-6 partitioning defective 6 homolog beta and FAM84B, family with sequence similarity 84, member B.

DACH1-regulated genes identified using gene expression analysis [17] were compared with p53-regulated genes. Three gene expression microarray datasets profiling DACH1 responsive genes were used for analysis (DACH1.0hr, DACH1.18h, DACH1.36h [17], mRNA). The function of genes that are regulated by either DACH1 or p53 were assessed (Fig. 1A,B). Functional enrichment analysis using pathways obtained from the molecular signatures database indicated a set of common pathways regulated by both p53 and DACH1 (Fig. 1A,B). We next examined the proportion of genes regulated by both DACH1 and p53 at serial time points. A total of 242, 2794, and 1621 DACH1-responsive genes were identified from the DACH1.0hr mRNA, DACH1.18h, and DACH1.36h datasets, respectively. Of these genes, 20 out of 242, 129 out of 2794, and 68 out of 1621 were also regulated by p53 (Fig. 1C).

We next examined occupancy of p53 and DACH1 in the genome by comparing ChIP-Seq. Sets of genes bound by p53 and DACH1 in breast cancer cells were obtained from ChIP-Seq data [18, 19]. For gene annotation, high-confidence ChIP-Seq regions were matched to the nearest proximal Ensemble gene identifier. A total of 1304 binding sites for p53 and 1844 binding sites for DACH1 were identified. 95 genes bound by both DACH1 and p53 were identified (p = 1.87 × 10^−14^, Fig. 1D, Table S1).

Distributions of the locations of DACH1 and p53 binding sites detected by ChIP-Seq demonstrated binding of active chromosomal regions proximal to gene coding regions, consistent with a model in which DACH1 and p53 localize to both very distal elements and promoter regulatory elements (Fig. 1E). The tag density profiles for p53 and DACH1 demonstrated a similar distribution of genomic association, comparing locations at the promoter, within a gene or downstream of the transcriptional stop site, with a relatively greater enrichment for p53 chipSeq peaks at more distal locations. A higher level resolution of relative binding in ChIP-Seq of p53 and DACH1 in relation to the transcriptional start site (TSS) confirmed this observation (Fig. 1F). This may suggest that DACH1 and p53 co-regulation occurs at distal regulatory sites. Figure 1G shows the tag density of DACH1 and p53 chipSeq peaks relative to the coding regions of two co-regulated genes, PARD6B and FAM84B. These figures demonstrate that co-localization of DACH1 and p53 occurs proximal to the promoter region for at least a subset of genes.

### DACH1 enhances p53-mediated cell-cycle arrest and apoptosis

**Figure 2:**
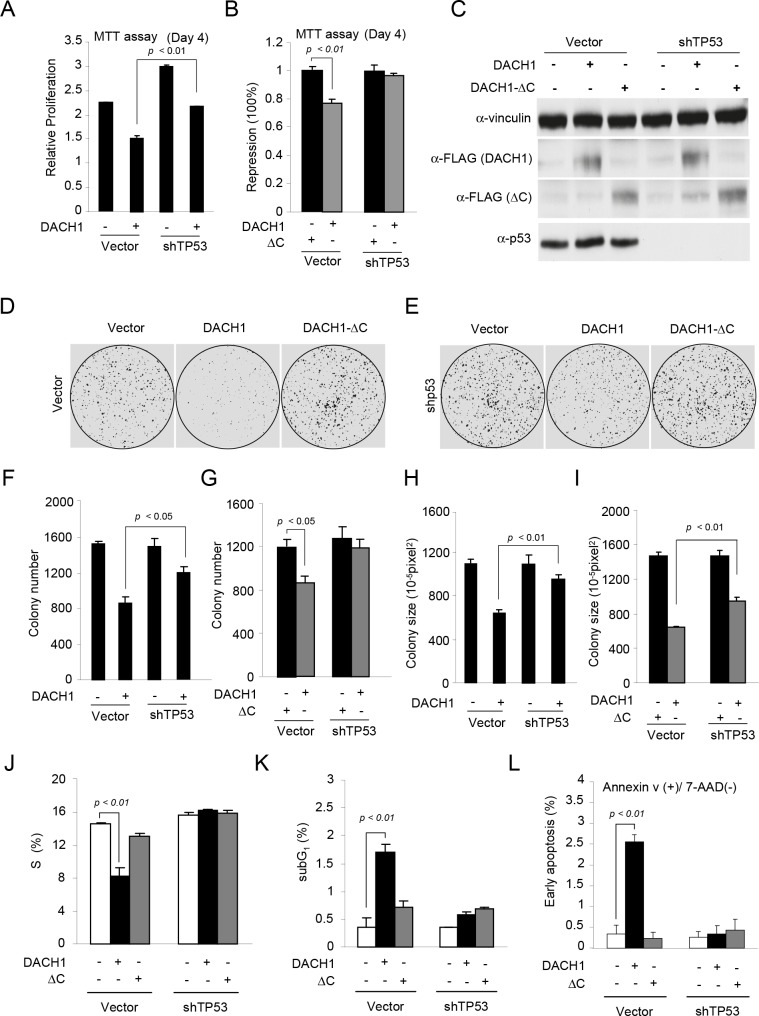
DACH1 enhances p53-dependent inhibition of colony formation and induction of apoptosis (A-B) MTT assay of proliferation in MCF-7 cells expressing either DACH1 or DACH1 ΔC in the presence of either control or shRNA to p53. (C) Western blot for p53 and DACH1 (identified by the FLAG tag). (D-I) Colony formation assays using MCF-7 cells expressing either wild-type DACH1 or ΔC in the presence of control or shRNA to p53 (D,E), representative examples of colonies formed and (F-I) quantification of data shown as mean ± SEM. In (J-L) analysis of MCF-7 cells stably expressing DACH1 and shp53 for (J) S-phase of the cell-cycle (K), sub-G_1_ and (L) Annexin V staining as a marker of apoptosis.

In order to examine the functional significance of p53 in DACH1-dependent function, MCF-7 cells were stably transduced with either DACH1 or a DACH1 mutant that was defective in p53 binding (DACH1 ΔC) and then sequentially transduced with p53 shRNA (Fig. 2). DACH1 inhibited cell proliferation assessed using the MTT assay. p53 shRNA increased proliferation, and the ability of DACH1 to inhibit proliferation was reduced by p53 shRNA (Fig. 2A). DACH1 expression reduced cellular proliferation, requiring the carboxyl terminus(Fig. 2B). shRNA to p53 abrogated DACH1 repression, demonstrating the inhibition of proliferation by DACH1 is p53-dependent. To examine the role of the p53 binding domain of DACH1, the DACH1 carboxyl terminal deletion mutant (DACH1 ΔC) was deployed. WB was conducted of the cells to confirm the reduction of p53 levels with p53 shRNA and the levels of expression of DACH1 ΔC compared with DACH1 WT (Fig. 2C). DACH1 abundance assessed by the FLAG epitope showed similar levels of DACH1 WT vs. DACH1 ΔC. The p53 shRNA treated cells showed reduced levels of p53 protein (Fig. 2C).

The role of p53 in DACH1-mediated suppression of contact-dependent growth was assessed in MCF-7 cells. p53 shRNA reduced DACH1-mediated inhibition of colony formation (Fig. 2D-F). Deletion of the DACH1 carboxyl terminus reduced the inhibition of colony formation both in size and number (Fig. 2F-I). DACH1 reduced the proportion of cells in the S-phase of the cell-cycle, which was abolished by deletion of the DACH1 carboxyl terminus. The inhibition of S-phase was p53-dependent, as it was abrogated by p53 shRNA. DACH1 expression induced the sub-G_0_ phase, requiring the DACH1 C-terminus and was abolished by p53 shRNA (Fig. 2K). The induction of apoptosis by DACH1 assessed by Annexin V staining, also required the DACH1 C-terminus and endogenous p53 (Fig. 2L). DACH1 inhibited the proportion of cells in S-phase, which was abolished by deletion of the DACH1 C-terminus. p53 shRNA abrogated the DACH1-mediated inhibition of S-phase.

### NAD-dependent acetylation determines DACH1 association with p53

**Figure 3:**
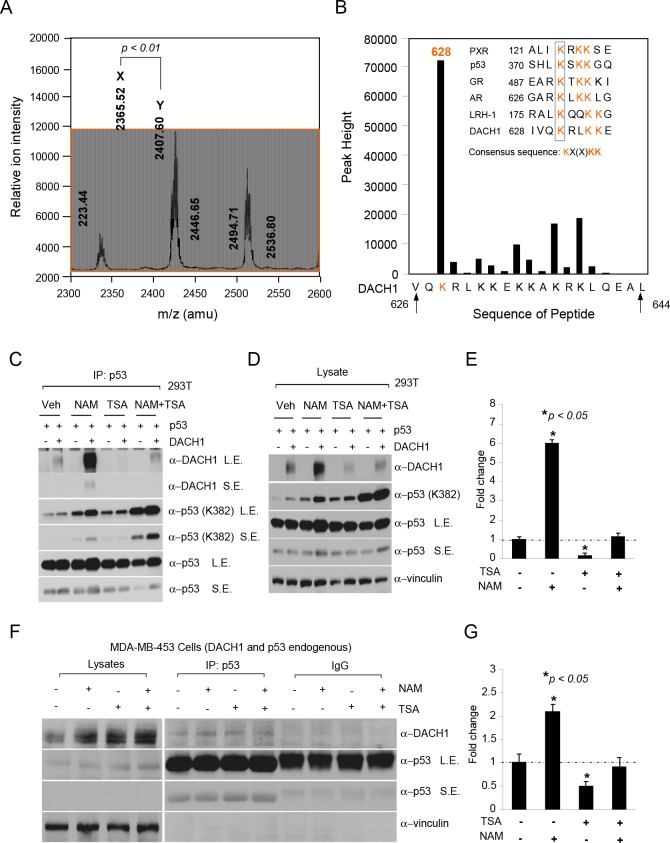
NAD-dependent binding of DACH1 to p53 (A) A peptide encoding the candidate DACH1 acetylation site (626 VQKRLKKEKKAKRKLQEAL 644) was incubated with p300 as an enzyme source in the presence of acetyl CoA. Acetylation products were purified with reverse-phase HPLC followed by MALDI-TOF mass spectrometry (A) and then sequenced by Edman degradation. In (A), the resulting DACH1 peptide (DACH1aa 626-644) mass spectrum is shown with mass/charge expressed in atomic mass units (amu). The major peaks labeled X corresponds to the expected mass of DACH1 whereas the large peak Y, is larger by 42 mass units, representing a single acetylated peptide. In (B) the bars represent the amount of phenylthiohydantoin-acetyl-lysine present in the corresponding position. The major acetylated product corresponds to residue K628. The inset shows an alignment of the DACH1 sequence and the acetylated residue motif seen in other acetylated transcription factors and the corresponding consensus sequence KX(x)KK. (C) IP-WB of HEK293T cells transfected with expression vectors encoding p53 or DACH1 and subsequently treated with Nicotinamide (NAM 20 mM) and/or Trichostatin A (TSA 2μm) for 8hrs. (D) Western blot of lysates corresponding to IP-Western in (C). (E) Relative binding of DACH1 to p53 shown as mean ± SEM for 3 separate experiments. IP-Western blot of MDA-MB-453 cells treated with TSA (2 μM) or NAM (20 mM) showing (G) relative binding of p53 to DACH1 (data are mean ± SEM for 3 separate experiments).

Previous studies have demonstrated the physical association of DACH1 with the acetyl-transferase CBP [20], and in chromatin immunoprecipitation (ChIP) and WB, the association of DACH1 with SIRT1 and HDAC [6]. Collectively, these studies suggested a role for DACH1 in binding to decetylases. In order to determine whether DACH1 could serve as a substrate for acetylates and decetylases, we conducted a mass spectrometry analysis of the DACH1 protein. LC/MS analysis of DACH1 after trypsin digestion identified peptides with high mascot scores. The panel of peptides mapped acetylation sites to residues Lysine 628. In order to determine whether lysine residue K628 within the 628-633 motif was acetylated, Edman-degradation analysis was conducted revealing acetylation, primarily of Lysine 628 (Fig. 3A). The DACH1 Lys 628 resembles a core acetylation motif found in p53 and nuclear receptors (Fig. 3B inset).

In order to determine whether the association between DACH1 and p53 was regulated by NAD- or TSA-sensitive HDACs, cells were treated with either Nicotinamide (20 mM) or TSA (2 μM) for 8 hours. Immuno-precipitation was conducted using an antibody to p53 with subsequent WB to DACH1 (Fig. 3C). The addition of NAD enhanced DACH1 binding to p53, 6-fold (Fig. 3C-E). In contrast, TSA reduced DACH1 binding to p53 (Fig. 3C-E). The importance of HDAC in the association between endogenous DACH1 and p53 was determined in MDA-MB-231 cells. As observed in 293T cells, nicotinamide enhanced binding (2-fold) and TSA reduced binding (Fig. 3F, G).

In order to determine whether the DACH1 acetylated residue affected p53 association, point mutation was conducted of the acetylated residue and mammalian expression vectors for the point mutants that were co-expressed with p53 in HEK293T cells.

### DACH1 augments p53-dependent transcription of DNA damage and cell-cycle arrest genes

We next examined the functional significance of DACH1 on p53-dependent expression of the known target genes p21^CIP1^ and RAD51. p53 bound the p21^CIP1^ regulatory region in ChIP-Seq. In MDA-MB-231 cells p21^CIP1^ was induced 1.6-fold and RAD51 was repressed 3.3-fold. In MCF-7 cells p21^CIP1^ was induced 3-fold and RAD51 was repressed 1.8-fold. The DACH1-deficient MCF-7 cell line was used, in which DACH1 was stably transduced. In order to determine the mechanism by which DACH1 induced p21^CIP1^ and reduced RAD51 abundance, we examined the ability of DACH1 to regulate their transcription using the promotor-regulatory region of these two target genes assessed in luciferase reporter assays. DACH1 expression inhibited RAD51 reporter activity (S. 4A). A DACH1 mutant deleted of the C-terminus failed to repress RAD51 reporter activity (S. 4B).

p53 shRNA was used to stably reduce p53 levels in MCF-7 cells, in order to examine the re-expression of either p53 or a p53 mutant that evades defective DACH1 binding. The re-expression of p53 enhanced p21^CIP1^ promoter activity. Re-expression of the p53 mutant R248Q reduced p21^CIP1^ (S. 4C). DACH1 enhanced p53 wt dependent activity of the p21^CIP1^ promoter. Transduction of MCF-7 cells with the DACH1 binding defective mutant p53-R248Q mutant did not enhance p21^CIP1^ transcription and DACH1 did not affect p21^CIP1^ promoter activity in the presence of p53-R268Q, suggesting the effect of DACH1 on p21^CIP1^ required p53 association. These findings are consistent with the observation that DACH1 is defective in binding the R248Q mutant. DACH1 enhancement of p53-dependent induction of p21^CIP1^ required the DACH1 C-terminus (S. 4D). DACH1 expression was sufficient to induce the activity of multimeric p53-response element and deletion of the C-terminus reduced p53 activity ~50% (S. 4E). DACH1 is known to bind a Forkhead like binding site [4]. DACH1 repression of a DACH1 response element in MCF-7 cells, an effect that was abrogated by p53 shRNA (S. 4E,F).

### DACH1 and RAD51 expression are inversely correlated in Luminal A and Basal human breast cancer

**Figure 4:**
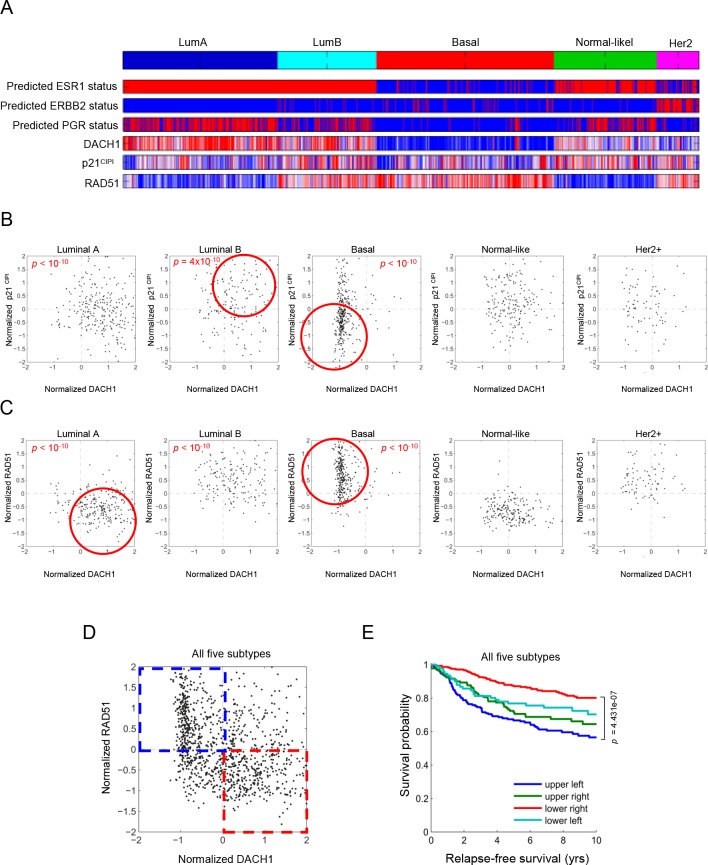
Subtype classification of breast cancer microarray samples Heatmap depicting samples from combined breast cancer microarray datasets that were assigned to breast cancer gene expression subtypes. The ESR1 (ERα), epidermal growth factor receptor 2, (ErbB2) and progesterone receptor (PR) mRNA status is shown, together with relative abundance of DACH1, p21^CIP1^ (B) and RAD51 (C). The luminal (A and B) and basal breast cancer subtypes are outlined (red circle) for DACH1 vs. p21^CIP1^ or DACH1 vs. RAD51 abundance. A significant positive correlation between DACH1 and p21^CIP1^ is shown for luminal B (p=4×10^−10^) and basal breast cancer (p=1×10^−10^). A significant inverse correlation is shown for DACH1 and RAD51 in luminal A (p=1×10^−10^) and basal breast cancer (p<10^−10^). (D) A depiction of relative abundance of RAD51 and DACH1 for all breast cancer and (E) Kaplan-Meier plot showing differences in metastasis-free survival. The patients in which DACH1 and RAD51 expression remain inversely correlated showed improved survival (P<4.43×10^−7^).

In view of the finding that DACH1 induced p21^CIP1^ and repressed RAD51, via p53, we investigated whether this gene expression relationship occurred in human breast cancer. We interrogated a data set of >2,250 annotated human breast cancer samples (Fig. 4A). The relative abundance of DACH1 and p21^CIP1^ were compared amongst the five distinct mRNA subtypes of human breast cancer (Fig. 4B). Consistent with the finding that DACH1 induced p21^CIP1^ via p53, DACH1 and p21^CIP1^ abundance was positively correlated in luminal B (p = 4×10^−10^) and basal breast cancer (p<10^−10^)(Fig. 4C). Furthermore, when all breast cancer tumor types were considered together, patients with tumors in which DACH1 expression was increased with a corresponding decrease in RAD51 levels (red square Fig. 4D), had improved relapse-free survival in Kaplan-Meier analysis (Fig. 4E).

## DISCUSSION

The studies reported here demonstrate that p53 binds to the cell-fate determination factor, DACH1. Mutational analysis demonstrated the specificity of binding by identifying the carboxyl terminus of DACH1 and key amino acids of p53 required for binding. p53 mutations occur in ~25% of human breast cancer. Herein, the p53-P72R and p53-R273H evaded DACH1 binding. The p53 polymorphism P72R showed reduced DACH1 binding. The P72R polymorphism occurs in a proline rich region of p53 known to be important for growth suppression and apoptotic functions [19]. The P72R showed reduced ability to induce programmed cell death and reduced ubiquitination and nuclear export [21-23]. The R248Q and R273H are hot spot mutations that arise in human cancer and are classified as a “contact” mutant, in which the overall architecture of the DBD is retained, but critical DNA contact points are lost [24]. DACH1 inhibits metastasis and R273H mutant knock-in mice show increased metastasis [25], raising the possibility that evasion of DACH1 binding may contribute to the “gain of function” by the R273H mutant. The cell-cycle arrest phenotype of p53 depends on the ability to induce the transcription of p21. Herein, DACH1 inhibition of S-phase and p21 transcription required p53 and the C-terminal DACH1 p53 binding domain.

DACH1 induced apoptosis via p53. The ability of p53 to induce apoptosis plays an important role in tumor suppression [26, 27]. The induction of apoptosis by p53 involves a distinct class of genes, including BAX, PUMA, NOXA and PIG3, which were also induced by DACH1 [17]. Given that a significant proportion of genes bind both DACH1 and p53 in ChIP-Seq, the mechanism by which DACH1 enhances p53-dependent function may involve induction of common target genes through either common or distinct CIS elements. DACH1 was capable of enhancing both DACH1 activity through the FOXO-like element and via p53 dependent transactivation.

Herein, the NAD-dependent HDAC, deacetylase SIRT1 enhanced DACH1/p53 association. DACH1 enhanced p53-dependent transactivation and DACH1-dependent transcription via a DACH1 response element in a p53-dependent manner. Thus, DACH1/p53 forms a signaling module, which is augmented by SIRT1. Acetylation of p53 can negatively regulate protein-protein interactions, and unacetylated p53 binds more strongly to Mdm2 and Mdmx [11]. The DACH1/p53 module is characterized by overlapping gene sets and function. DACH1 was shown to be acetylated, with K628 being identified by LC/MS and Edman degradation. Mutation of DACH1 K628 to create residues that could not gain an acetyl group, enhanced p53 binding. DACH1 showed enhanced binding to p53-K120R. In contrast, mutation of the carboxyl-terminal nine lysine residues, abolished p53-dependent induction of p21 without affecting MDM2 binding. The K120R mutant is competent for cell-cycle arrest and binding to p21 and hMDM2 promoters, while being defective for apoptosis [14, 16]. p53 K120 is acetylated via Tip60/hMOF and is a recurrent target for mutation during tumor development [14, 28]. These findings are consistent with a model in which distinct p53 lysine residues coordinate dissociable functions and suggest that the DACH1/p53 module engages K120 [29, 30]. As SIRT1 is known to bind p53 in the nucleus and deacetylate p53 at least at K382 and considering this residue is also deacetylated by HDAC [31], it would be of interest to determine whether the reduction in p53 associated with DACH1 in NAM with TSA correlates with an increase in acetylated p53 at K382. Treatment with the SIRT inhibitor nicotinamide enhanced the relative amount of DACH1 bound to p53, whereas the HDAC I and II inhibitor, TSA, did not. These findings suggest NAD-dependent deacetylation of DACH1 augments p53 association.

The decreased bindings of DACH1 to the p53 phosphorylation defective mutants are consistent with prior findings that DACH1 associates with CBP [20]. Phosphorylation of amino-terminal sites in p53, including Ser15 and Ser20, increases the association of p53 with p300/CBP and stimulates p53 transactivation [32, 33]. Phosphorylation of Ser15 is necessary for sequential modification, including acetylation of lysine residues in the p53 DBD and carboxyl terminal domains (reviewed in [33]). Phosphorylation of these residues also reduces MDM2 binding [34]. As Ser15 serves at the distal arm of DNA damage and stress signaling pathways, along with ATM/ATR and DNA-PK [35, 36], these studies raise the possibility that DACH1 may function to inhibit DNA damage signaling. It will be of interest to determine whether DACH1 is capable of inhibiting other DNA damage signaling pathways, such as phosphorylation of H_2_AX.

## MATERIALS AND METHODS

### Immunohistochemistry

Immuno-histochemical analysis of human breast cancer cell lines was conducted using a polyclonal DACH1 antibody [7].

### Cell culture, plasmid construction, reporter genes, expression vectors, DNA transfection, and luciferase assays

Cell culture, DNA transfection, and luciferase assays using the Rad51-Luc and p21-Luc reporter genes were performed as previously described [37]. The HEK293T, MDA-MB-453, MDA-MB-231 and SKBR3 HEK293T cells were cultured in DMEM supplemented with 10% fetal calf serum, 1% penicillin, and 1% streptomycin and were previously described [7]. The expression plasmids encoding an N-terminal FLAG peptide linked to DACH1 or DACH1 deleted of the DNA binding domain (ΔDS) were previously described [4]. The expression vector encoding the DACH1 alternate splice forms were sub-cloned into p3xFLAG-CMV-10 vector (Sigma). The expression vectors encoding wildtype or mutant p53 in the vector pLKO.1 (shp53 sequence: 5' – AAACCCAGGGCTGCCTTGGAAAAG – 3') p53 shRNA expression vector were previously described [14, 38]. p53 shRNA transfection and infection followed standard protocols [5,6,7]. GFP positive cells were selected by FACS. Cells were plated at a density of 1 × 10^5^ cells in a 24-well plate on the day prior to transfection with Superfect according to the manufacturer's protocol (Qiagen, Valencia, CA). A dose-response was determined in each experiment with 50 and 200 ng of expression vector and the promoter reporter plasmids (0.5 μg). Luciferase activity was normalized for transfection efficiency using β-galactosidase reporters as an internal control. The -fold effect of expression vector was determined with comparison to the effect of the empty expression vector cassette and statistical analyses were performed using the t- test.

### Cell Proliferation Assays

Cells infected with MSCV-IRES-GFP, MSCV-DACH1-IRES-GFP, MSCV-DACH1ΔC or C-term-IRES-GFP, were seeded into 96 well plates in normal growth medium, and cell growth was measured daily by MTT assays using 3-(4, 5-dimethylthiazol-2-yl)-2, 5-diphenyltetrazoliumbromide.

### Colony forming assays

4 × 10^3^ cells were plated in triplicate in 3 ml of 0.3% agarose (sea plaque) in complete growth medium in the presence or absence of 2 μg/ml doxycycline overlaid on a 0.5% agarose base, also in complete growth medium. 2 weeks after incubation, colonies more than 50 μm in diameter were counted using an Omnicon 3600 image analysis system. The colonies were visualized after staining with 0.04% crystal violet in methanol for 1 to 2 h.

### Identification of DACH1 phosphorylation and acetylation sites by mass spectrometry and Edman degradation assays. Phosphopeptide mapping

DACH1 protein was isolated by immunoprecipitation and separated by gel electrophoresis. The gels were stained with Coomassie G250 and the bands were excised and digested with trypsin. Phosphorylated peptides were isolated using affinity purification using TiO2 Nu-tips from Glygen. Briefly, the extracted peptides were loaded on the tip in a buffer containing 300 mg/ml DHB in 80% Acetonitrile, 0.1% TFA, washed once with the loading buffer and once with 80% Acetonitrile, 0.1% TFA and eluted in 0.4M Ammonium Hydroxide. Peptides were immediately acidified with Formic Acid and were analyzed by ESI-MS/MS on a Thermo-Electron ProteomeX LC/MS workstation as previously described [39]. Spectra were searched against the Swissprot database using Mascot (Matrix Science) with Carbamidomethyl as a fixed modification and Oxidation (M), Phospho (ST), Phospho (Y) as variable modifications. Peptides that had a score greater than 45 were reported as statistically significant (p<0.05) [40].

Dehydrated peptides were re-suspended in 5% acetonitrile, 0.05% formic acid and immediately loaded on a nano-spray tip for LC-MS/MS analysis. 10 - 15% of the peptide digest is loaded on a Magic C18 AQ (Michrom) nanospray tip, packed to 5 cm. This tip was loaded, using a pressure bomb, and washed, after installation on the HPLC of a Thermo LTQ mass spectrometer, with 5% methanol, 0.1% formic acid, for 10 min with a flow rate of 600 nl/minute (about 10 column volumes = 6.6 μl) The peptides were eluted and analyzed in an LC-MS/MS run, using a 5-15% methanol gradient over 2.5 minutes, followed by a 15-60% methanol gradient for 67 minutes, a 60% methanol isocratic step of 4 minutes, ending with a 3-minute 95% methanol step, with all solvents containing 0.1% formic acid. A full MS survey scan was performed every 3 seconds and the top 7 peaks were selected to produce MS/MS fragmentation spectrum. To increase coverage of basic peptides, chymotrypsin digests were also applied to an LC-MS/MS run at neutral pH in 20 mM ammonium format, instead of 0.1% formic acid, using an identical gradient elution program and mass spectrometry run, as above.

### Mapping of proteolytic peptide fragments and acetylation sites

The MS and MS/MS fragmentation spectrum data were used in a Mascot search of the whole human proteome. To identify peptide sequences modified with acetyl groups, a custom database, containing the recombinant DACH1 sequence, was also searched. The following search criteria were used for selecting fragmentation spectra that map to proteolytic peptides: peptide tolerance = -0.8 to +0.5, a minimum ion score of 15, and a fragmentation spectrum, containing fragment ions that either include or flank the acetylated amino acid position. Mascot searches were conducted, allowing for multiple positive charge-states, 2, 3, or 4 missed cleavage sites, fixed S-carboxyamidomethyl modification of cysteine and variable methionine oxidation and lysine acetylation.

Edman degradation assays were conducted using an acetylated DACH1 peptide. The synthetic peptide corresponding to the DACH1 (residues a.a. 626-644, NH2-VQK RLK KEK KAK RKL QEAL-COOH) that contains lysine-rich motif was synthesized by Bio·Synthesis (Lewisville, TX) and purified to 95% purity by HPLC. The peptides were acetylated in vitro by incubation with 5 mM acetyl-CoA and baculovirus-purified FLAG-p300 at 30°C for 2h. After incubation, acetylated peptides were separated from contaminating p300 by passage through a micron filter (Amicon Inc., Beverly, MA) and further purified by analytical reversed phase HPLC. The reaction products were analyzed with a PE-Biosystems DE-STR MALDI-TOF mass spectrometer. Further analysis by Edman degradation was performed on a PE-Biosystems Procise sequencer and Phenylthiohydantoin-acetyl-lysine was measured by absorbance at 259 nm.

### Cell Cycle Analysis

Cell cycle parameters were determined using laser scanningcytometry. Cells were processed by standard methods usingpropidium iodide staining of cell DNA. Each samplewas analyzed by flow cytometry with a FACScan Flow Cytometer(Becton-Dickinson Biosciences, Mansfield, MA) using a 488 nmlaser. Histograms were analyzed for cell cycle compartmentsusing ModFit version 2.0 (Verity Software House, Topsham, ME).A minimum of 20,000 events was collected to maximize statisticalvalidity of the compartmental analysis. Apoptosis was determined by Annexin V staining [41].

### Immunoprecipitation and Western Blot

Immunoprecipitation (IP) and Western blot assays were performed in HEK293T cells as indicated. Cells were pelleted and lysed in buffer (50 mM HEPES, pH 7.2, 150 mM NaCl, 1 mM EDTA, 1 mM EGTA, 1 mM DTT, 0.1% Tween 20) supplemented with a protease inhibitor cocktail (Roche Diagnostics, Mannheim, Germany). Antibodies used for IP and Western blot were: anti-p53 (SC-126) and anti-FLAG (M2 clone, Sigma).

### Microarray and Cluster Analysis

DNA-free total RNA isolated from DACH1 inducible MDA-MB-231 cells were used to probe human OneArray (Phalanx). RNA quality was determined by gel electrophoresis. Analysis of the arrays was performed using GeneSpring. Arrays were normalized using robust multi-array analysis, and the p value of 0.05 was applied as a statistical criterion for differentially expressed genes. These genes were then grouped using hierarchical clustering with “complete” agglomeration, and each cluster was further analyzed based upon the known function of the genes contained in the cluster. Expression profiles are displayed using Treeview. Classification and clustering for pathway level analysis were performed by using gene sets ASSESS (Analysis of Sample Set Enrichment Scores), and DAVID available on line. ASSESS provides a measure of enrichment of each gene set in each sample.

### Genomic occupancy of DACH1 and p53. Identification of genes with DACH1 and p53 binding sites

Binding sites for p53 in breast cancer cells were obtained from a ChIP-Seq analysis of chromatin occupancy by p53 following activation by three different molecules, nutlin3a, RITA and 5-fluorouracil (5-FU) [18]. High-confidence ChIP-Seq peaks were identified as described [18] by applying the following filters: p≤0.05, ≥2 fold enrichment over IgG control, peak area ≥20. The intersections of peaks identified from the three p53 inducing treatments were used as p53 binding sites. DACH1 binding sites were identified from ChIP-Seq of a breast cancer cell line stably expressing DACH1 [4]. ChIP-Seq peaks were mapped to the nearest proximal Ensemble gene identifier. Significant overlap in p53 and DACH1 regulated genes was tested using the hypergeometric distribution with all ensemble gene identifiers in homo sapiens used as a reference set. Annotation of the location of ChIP-Seq peaks relative to gene coding regions was facilitated by the ChIPpeakAnno and GenomicRanges packages in Bioconductor. The Integrated Genome Browser software package was used for visualization of ChIP-seq tag density relative to gene coding regions.

### Identification of genes regulated by DACH1

Genes regulated by DACH1 were also identified from gene expression microarray experiments. In the first experiment, gene expression was measured in MDA-MB-231 cells engineered to express DACH1 and treated with either vehicle or ponasterone A for 18 and 36 hours. Differentially expressed genes were identified as genes with a 1.5 fold-change on average in ponasterone A treated vs. control DACH1-inducible MDA-MB-231 cells. Affymetrix probe set identifiers were mapped to Ensemble gene identifiers using information from Affymetrix annotation files. Significant overlap in p53- and DACH1- regulated genes was tested using the hypergeometric distribution with all Ensemble gene identifiers annotated on the Affymetrix chip as a reference set. In order to identify signaling pathways enriched with p53- or DACH1-regulated genes, the hypergeometric test was used with pathway gene sets derived from the molecular signatures database.

## SUPPLEMENTARY TABLES AND FIGURES




