# Supplementary material for: Acetylation of the Cell-Fate Factor Dachshund Determines p53 Binding and Signaling Modules in Breast Cancer
Source: Oncotarget. 2013 Jun 21;4(6):923–35. doi: 10.18632/oncotarget.1094 (PMC3757249; doi:10.18632/oncotarget.1094)
Supplement: Supplementary file 1 [file oncotarget-04-923-s001.doc]

Acetylation of the Cell-Fate Factor Dachshund Determines p53 Binding and Signaling Modules in Breast Cancer - Chen et al

**Supplemental Table 1: Genes regulated by both DACH1 and p53 assessed by ChipSeq**

| Gene ID | External gene ID | Entrezgene |
| --- | --- | --- |
| ENSG00000005175 | RPAP3 | 79657 |
| ENSG00000005884 | ITGA3 | 3675 |
| ENSG00000010292 | NCAPD2 | 9918 |
| ENSG00000011052 | NME2 | 654364 |
| ENSG00000011052 | NME2 | 4831 |
| ENSG00000041802 | LSG1 | 55341 |
| ENSG00000053254 | FOXN3 | 1112 |
| ENSG00000070882 | OSBPL3 | 26031 |
| ENSG00000072274 | TFRC | 7037 |
| ENSG00000076604 | TRAF4 | 9618 |
| ENSG00000080546 | SESN1 | 27244 |
| ENSG00000083857 | FAT1 | 2195 |
| ENSG00000087088 | BAX | 581 |
| ENSG00000088986 | DYNLL1 | 8655 |
| ENSG00000100731 | PCNX | 22990 |
| ENSG00000100796 | SMEK1 | 55671 |
| ENSG00000101040 | ZMYND8 | 23613 |
| ENSG00000101166 | SLMO2 | 51012 |
| ENSG00000101198 | NKAIN4 | 128414 |
| ENSG00000101247 | NDUFAF5 | 79133 |
| ENSG00000104823 | ECH1 | 1891 |
| ENSG00000105643 | ARRDC2 | 27106 |
| ENSG00000108175 | ZMIZ1 | 57178 |
| ENSG00000108604 | SMARCD2 | 6603 |
| ENSG00000111596 | CNOT2 | 4848 |
| ENSG00000112941 | PAPD7 | 11044 |
| ENSG00000115459 | ELMOD3 | 84173 |
| ENSG00000116016 | EPAS1 | 2034 |
| ENSG00000119318 | RAD23B | 5887 |
| ENSG00000120137 | PANK3 | 79646 |
| ENSG00000121083 | DYNLL2 | 140735 |
| ENSG00000124171 | PARD6B | 84612 |
| ENSG00000124208 | TMEM189-UBE2V1 | 387522 |
| ENSG00000124208 | TMEM189-UBE2V1 | 7335 |
| ENSG00000124222 | STX16 | 8675 |
| ENSG00000124635 | HIST1H2BJ | 8970 |
| ENSG00000125871 | C20orf72 | 92667 |
| ENSG00000125968 | ID1 | 3397 |
| ENSG00000126821 | SGPP1 | 81537 |
| ENSG00000132334 | PTPRE | 5791 |
| ENSG00000133247 | SUV420H2 | 84787 |
| ENSG00000135655 | USP15 | 9958 |
| ENSG00000136003 | ISCU | 23479 |
| ENSG00000136197 | C7orf25 | 79020 |
| ENSG00000137449 | CPEB2 | 132864 |
| ENSG00000137500 | CCDC90B | 60492 |
| ENSG00000138061 | CYP1B1 | 1545 |
| ENSG00000138600 | SPPL2A | 84888 |
| ENSG00000139218 | SCAF11 | 9169 |
| ENSG00000141568 | FOXK2 | 3607 |
| ENSG00000143367 | TUFT1 | 7286 |
| ENSG00000145632 | PLK2 | 10769 |
| ENSG00000148400 | NOTCH1 | 4851 |
| ENSG00000148690 | FRA10AC1 | 118924 |
| ENSG00000150676 | CCDC83 | 220047 |
| ENSG00000151743 | AMN1 | 196394 |
| ENSG00000155363 | MOV10 | 4343 |
| ENSG00000158555 | GDPD5 | 81544 |
| ENSG00000161714 | PLCD3 | 113026 |
| ENSG00000163882 | POLR2H | 5437 |
| ENSG00000165891 | E2F7 | 144455 |
| ENSG00000166262 | C15orf33 | 196951 |
| ENSG00000166886 | NAB2 | 4665 |
| ENSG00000167996 | FTH1 | 2495 |
| ENSG00000168209 | DDIT4 | 54541 |
| ENSG00000168214 | RBPJ | 3516 |
| ENSG00000168264 | IRF2BP2 | 359948 |
| ENSG00000168672 | FAM84B | 157638 |
| ENSG00000170312 | CDK1 | 983 |
| ENSG00000170734 | POLH | 5429 |
| ENSG00000171132 | PRKCE | 5581 |
| ENSG00000171469 | ZNF561 | 93134 |
| ENSG00000171617 | ENC1 | 8507 |
| ENSG00000172663 | TMEM134 | 80194 |
| ENSG00000172667 | ZMAT3 | 64393 |
| ENSG00000173334 | TRIB1 | 10221 |
| ENSG00000173744 | AGFG1 | 3267 |
| ENSG00000173914 | RBM4B | 83759 |
| ENSG00000173950 | XXYLT1 | 152002 |
| ENSG00000174579 | MSL2 | 55167 |
| ENSG00000175592 | FOSL1 | 8061 |
| ENSG00000177106 | EPS8L2 | 64787 |
| ENSG00000177200 | CHD9 | 80205 |
| ENSG00000177595 | PIDD | 55367 |
| ENSG00000178209 | PLEC | 5339 |
| ENSG00000180900 | SCRIB | 23513 |
| ENSG00000181610 | MRPS23 | 51649 |
| ENSG00000182095 | TNRC18 | 84629 |
| ENSG00000183048 | SLC25A10 | 1468 |
| ENSG00000184787 | UBE2G2 | 7327 |
| ENSG00000198824 | CHAMP1 | 283489 |
| ENSG00000199004 | MIR21 | 406991 |
| ENSG00000213123 | TCTEX1D2 | 255758 |
| ENSG00000221315 | MIR1204 | 100302185 |
| ENSG00000221763 | MIR1289-1 | 100302125 |
