# Supplementary material for: Acetylation of the Cell-Fate Factor Dachshund Determines p53 Binding and Signaling Modules in Breast Cancer
Source: Oncotarget. 2013 Jun 21;4(6):923–35. doi: 10.18632/oncotarget.1094 (PMC3757249; doi:10.18632/oncotarget.1094)
Supplement: Supplementary file 2 [file oncotarget-04-923-s002.pdf]

Acetylation of the Cell-Fate Factor Dachshund Determines p53 Binding and Signaling Modules in Breast Cancer - Chen et al

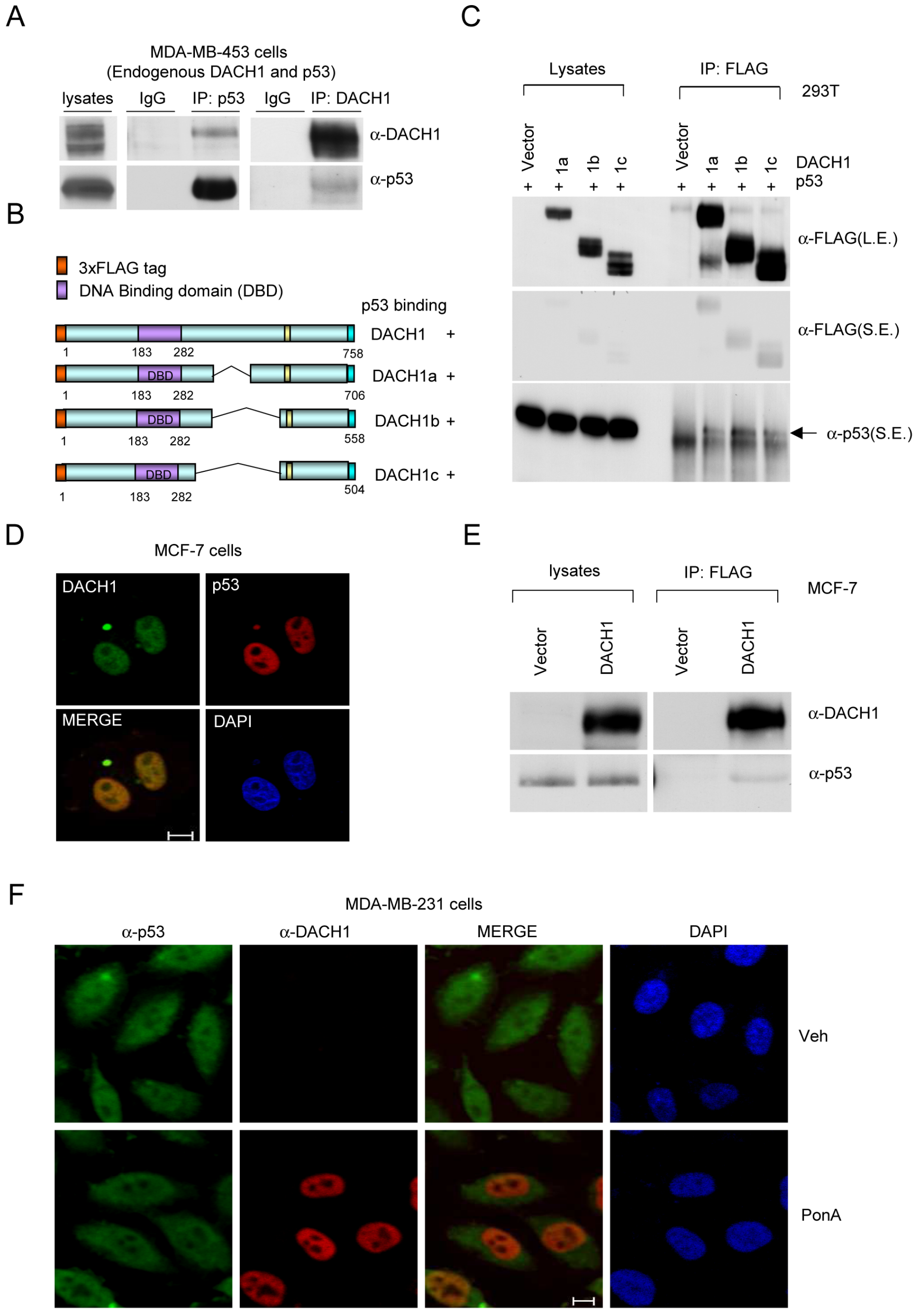

Supplemental Figure 1: DACH1 binds p53 in breast cancer cells. (A) Immuno-precipitation (IP) WB was conducted of endogenous DACH1 and p53 in MDA-MB-453 cells using antibodies as indicated. (B) Schematic representation of DACH1 protein and its alternatively spliced forms. (C) p53 and DACH1 binding determined by IP-WB analysis derived from HEK293T cells transfected with expression vectors for DACH1 and p53. (D) Immunofluorescent (IF) staining of MCF-7 cells for p53 (red), DACH1 (green) and nucleus (DAP1 – blue) showing co-localization of DACH1 and p53 (yellow) in the nucleus of MCF-7 cells. (E) IP –WB of MCF-7 cells in which FLAG-tagged DACH1 was used to co-precipitate p53. (F) MDA-MB-231 cells stably expressing a ponasterone-inducible FLAG-tagged DACH1 expression vector were treated with ponasterone for 24 hours (2 μM) and assessed by IF.

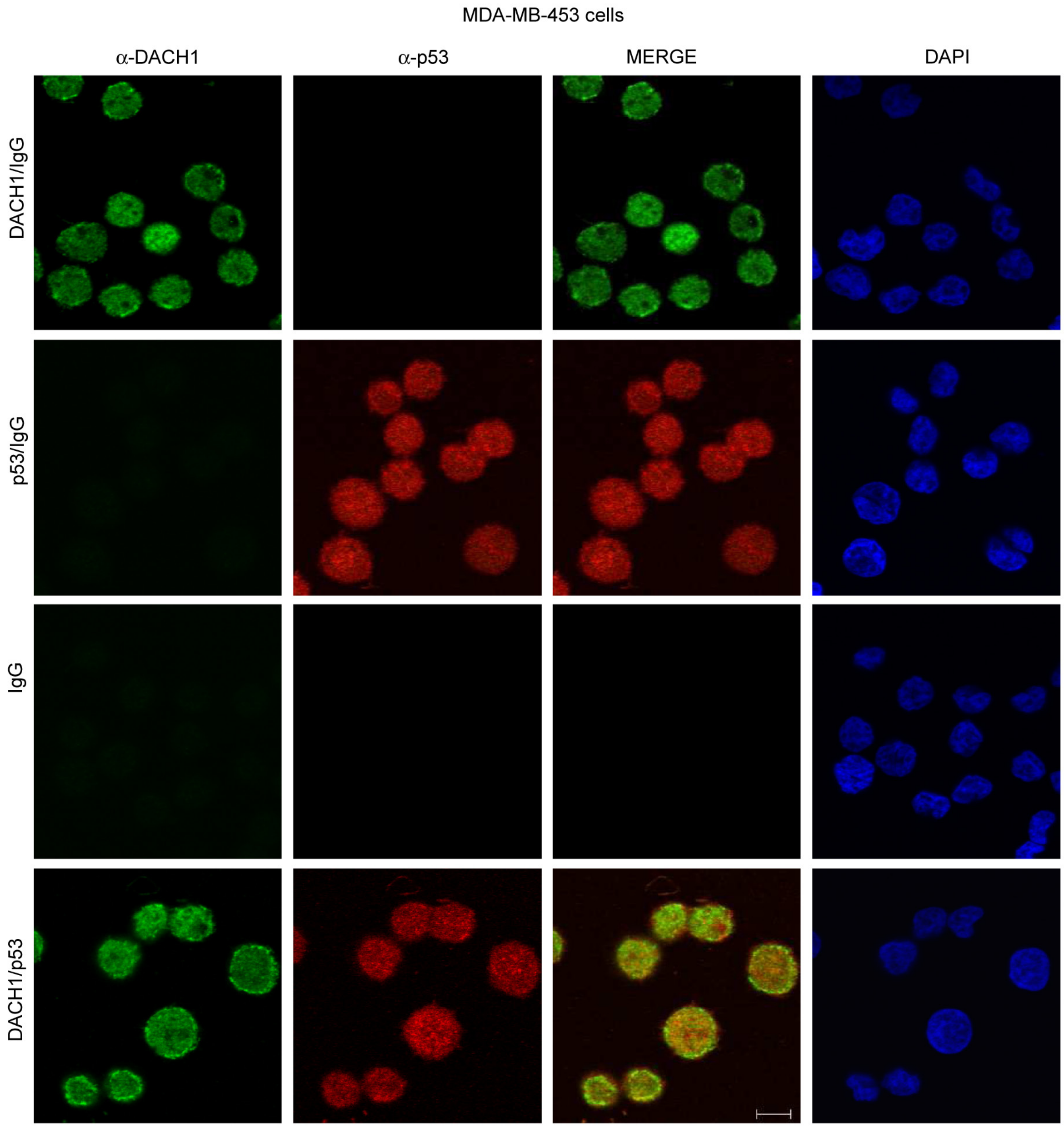

Supplementary Figure 2: DACH1 and p53 colocalize in breast cancer cells. Confocal microscopy images of p53 and DACH1 in MDA-MB-433 with merged images illustrating sites of colocalized proteins. (A) Schematic representation of DACH1 and p53 with the sites of interaction mapped in the current studies. (B) p53 functional domains and sites of post translational modifications co-acetylation, phosphorylation and the effect of those residues on the DACH1 binding.

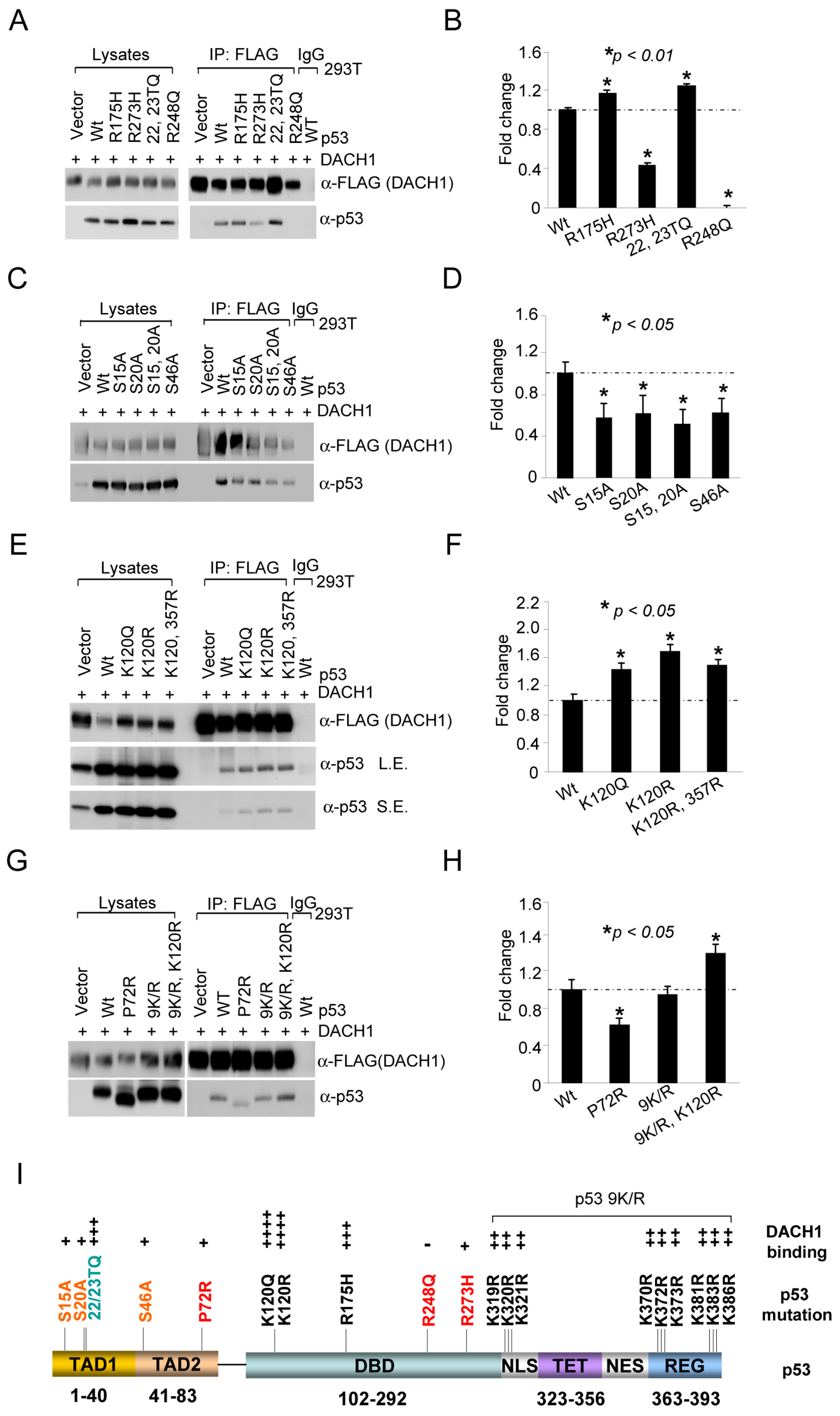

Supplemental Figure 3: p53 mutations in breast cancer evade DACH1 binding. (A-H) IP-WB of DACH1 binding to p53 mutants. HEK293T cells were transfected with the indicated expression plasmids and IP-WB was conducted. The relative binding of p53 to DACH1 was determined as mean ± SEM of N>3 separate transfections. A representative example of each experiment is shown. The p53 mutants are (B) mutations found in breast cancer, (D) phosphorylation site mutations (E-H) acetylation site mutants. (I) shows a schematic representation of p53 residues evaluated or tested for DACH1 binding.

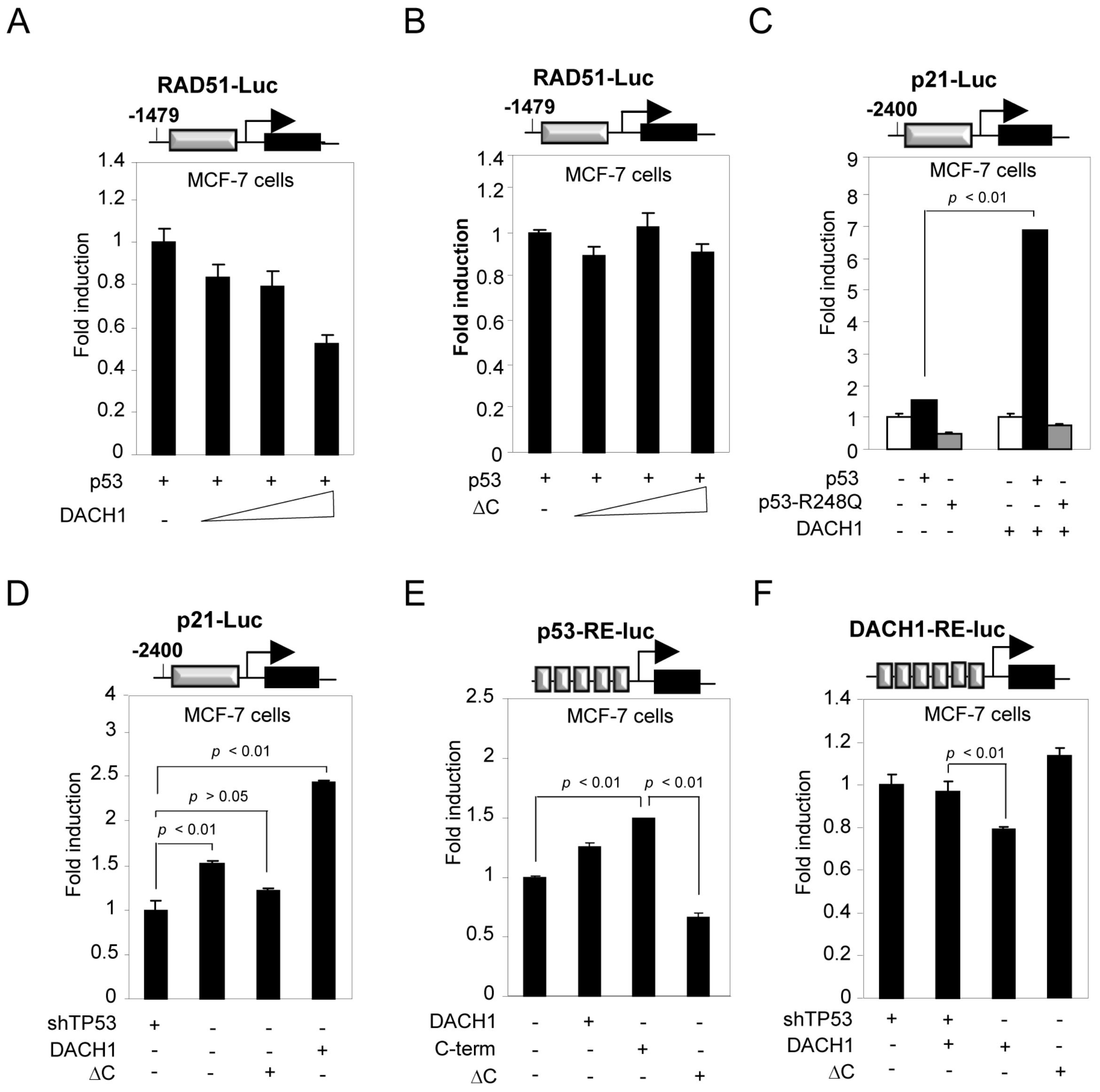

Supplemental Figure 4: DACH1 enhances p53-dependent transcriptional regulation of RAD51 and p21CIP1. The luciferase reporter assays were conducted using either the RAD51 or p21^{CIP1} promotor-luciferase reporters (A-D) or the synthetic response element for p53 (F) or DACH1. Cells were transduced with expression vectors as indicated and a p53 shRNA. The relative fold-induction of luciferase activity was normalized to shRNA vector and to *β*-galactosidase and shown as -fold change (data are mean ± SEM for N>5 separate experiments).

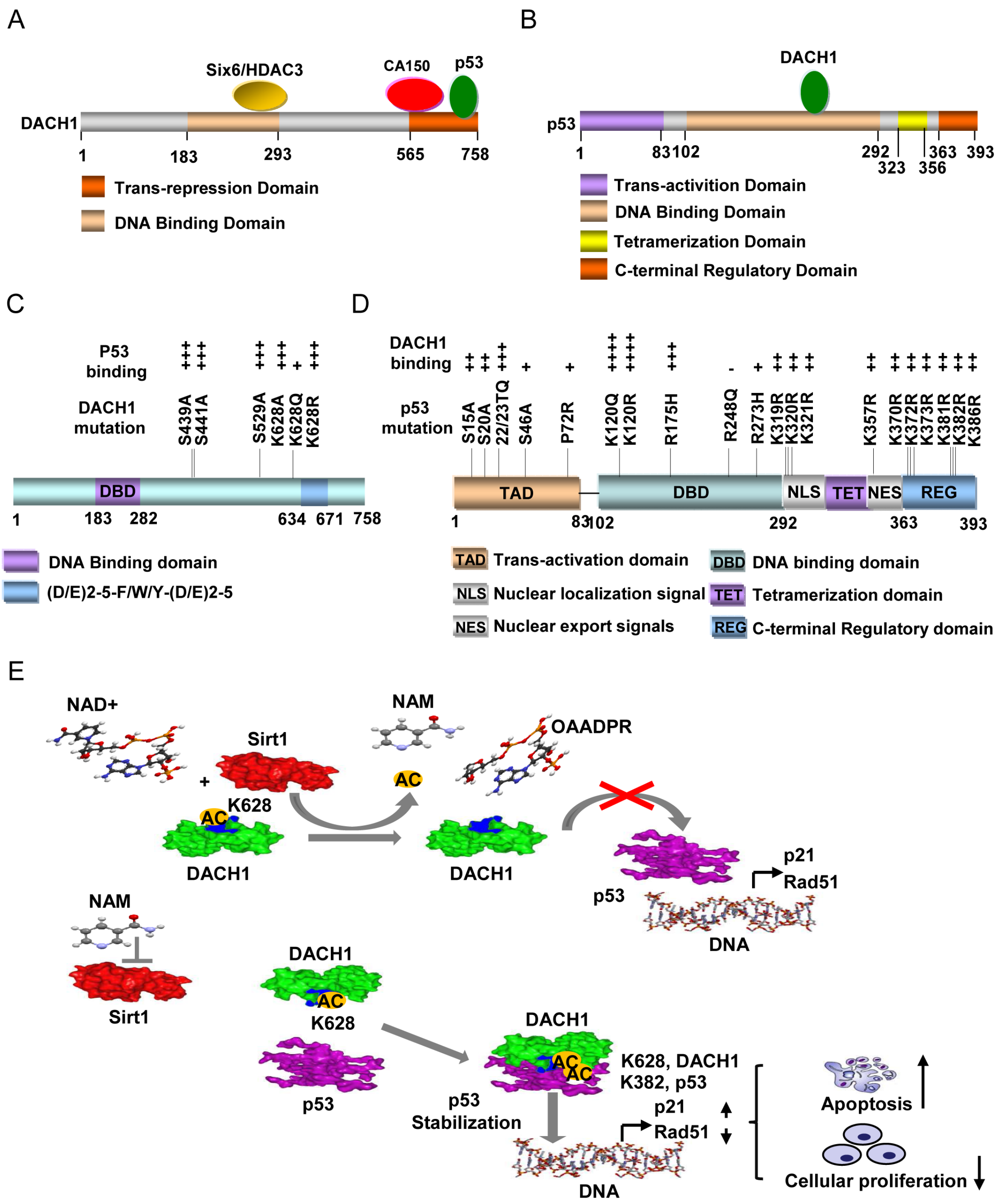

Supplemental Figure 5: Schematic representation of mechanisms by which Nicotinamide regulates association of DACH1 and p53. (A) DACH1 and its binding proteins including p53. (B) p53 protein and its functional domains that determine DACH1 association. (C) DACH1 and (D) p53 posttranslational modifications and their effect on protein association. (E) Model illustrating DACH1 deacetylation by SIRT1 uncoupling p53-mediated regulation of p21[CIP1] and RAD51. The acetylated carboxyl-terminal domain of DACH1 is required to bind and stabilize p53 in promoting apoptosis and cellular proliferation. Dachshund homolog 1 (DACH1, green molecule) is a nuclear factor essential for determining cell fate. DACH1 is deacetylated by the NAD-dependent histone decetylase enzyme (SIRT1, red molecule) on amino acid residue K628, located in the DACH1 carboxyl-terminal domain. When SIRT1 is inhibited (i.e. by nicotinamide, NAM), the deacetylation of DACH1 on K628 is inhibited and the acetylated form of DACH1 binds p53 (violet molecule). This interaction is mediated by the DACH1 carboxyl-terminal domain (blue domain, from aa 565 to aa 706). The binding of DACH1 to p53 stabilizes the complex and promotes p21[CIP1] expression and RAD51 down-regulation. The p53/DACH1 module increases apoptosis and inhibits cellular proliferation (NAD: Nicotinamide Adenine Dinucleotide, OAADPR: 2'-O-acetyl-ADP-ribose. The protein structures were downloaded from RCSB Protein Data Bank (http://www.rcsb.org/pdb/home/home.do).
